# *catena*-Poly[(dichloridocadmium)-di-μ-chlorido-[bis­(morpholinium-κ*O*)cadmium]-di-μ-chlorido]

**DOI:** 10.1107/S160053681102914X

**Published:** 2011-07-23

**Authors:** Wen-xiang Wang, Jing Dai

**Affiliations:** a Ordered Matter Science Research Center, College of Chemistry and Chemical, Engineering, Southeast University, Nanjing 211189, People’s Republic of China

## Abstract

In the title compound, [Cd_2_Cl_6_(C_4_H_10_NO)_2_]_*n*_, the coordination geometry of each Cd^II^ ion is distorted octa­hedral, but with quite different coordination environments. One Cd^II^ atom is coordinated by four Cl atoms and two O atoms from two morpholinium ligands, while the other is coordinated by six Cl atoms. Adjacent Cd^II^ atoms are inter­connected alternately by paired chloride bridges, generating a chain parallel to the *a* axis. Inter­chain N—H⋯Cl inter­actions form a two-dimensional network.

## Related literature

For general background to one-, two- and three-dimensional coordination polymers, see: Xiong *et al.* (1999 ▶); Ye *et al.* (2005 ▶); Zhao *et al.* (2008 ▶). For the dimeric coordination compound [(MOR)_2_Cu_2_Cl_6_] (MOR = morpholinium), see: Willett *et al.* (2005 ▶). 
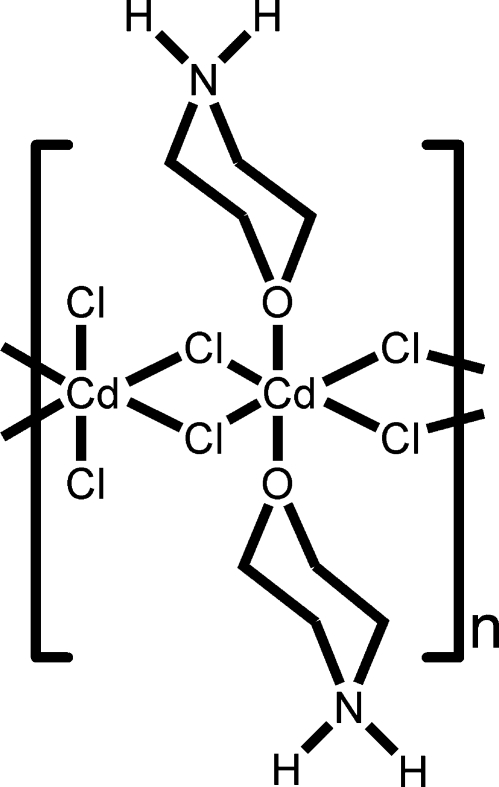


## Experimental

### 

#### Crystal data

[Cd_2_Cl_6_(C_4_H_10_NO)_2_]
*M* *_r_* = 613.78
Orthorhombic, 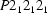

*a* = 7.0496 (14) Å
*b* = 14.404 (3) Å
*c* = 17.583 (4) Å
*V* = 1785.4 (7) Å^3^
*Z* = 4
Mo *K*α radiation
μ = 3.28 mm^−1^
*T* = 298 K
0.45 × 0.30 × 0.15 mm

#### Data collection

Rigaku SCXmini diffractometer
Absorption correction: multi-scan (*CrystalClear*; Rigaku, 2005 ▶) *T*_min_ = 0.319, *T*_max_ = 0.611
18533 measured reflections
4100 independent reflections
3893 reflections with *I* > 2σ(*I*)
*R*_int_ = 0.035

#### Refinement

*R*[*F* ^2^ > 2σ(*F* ^2^)] = 0.023
*wR*(*F* ^2^) = 0.051
*S* = 1.07
4100 reflections
181 parameters
H-atom parameters constrained
Δρ_max_ = 0.31 e Å^−3^
Δρ_min_ = −0.61 e Å^−3^

### 

Data collection: *CrystalClear* (Rigaku, 2005 ▶); cell refinement: *CrystalClear*; data reduction: *CrystalClear*; program(s) used to solve structure: *SHELXS97* (Sheldrick, 2008 ▶); program(s) used to refine structure: *SHELXL97* (Sheldrick, 2008 ▶); molecular graphics: *SHELXTL* (Sheldrick, 2008 ▶); software used to prepare material for publication: *SHELXTL*.

## Supplementary Material

Crystal structure: contains datablock(s) I, global. DOI: 10.1107/S160053681102914X/jh2310sup1.cif

Structure factors: contains datablock(s) I. DOI: 10.1107/S160053681102914X/jh2310Isup2.hkl

Additional supplementary materials:  crystallographic information; 3D view; checkCIF report

## Figures and Tables

**Table 1:** Hydrogen-bond geometry (Å, °)

*D*—H⋯*A*	*D*—H	H⋯*A*	*D*⋯*A*	*D*—H⋯*A*
N1—H1*A*⋯Cl5^i^	0.90	2.52	3.203 (3)	133
N1—H1*A*⋯Cl6^ii^	0.90	2.98	3.733 (4)	143
N1—H1*B*⋯Cl5^iii^	0.90	2.38	3.183 (3)	149
N2—H2*C*⋯Cl3^iv^	0.90	2.56	3.276 (3)	137
N2—H2*C*⋯Cl2^iv^	0.90	2.76	3.323 (3)	122
N2—H2*D*⋯Cl3^v^	0.90	2.73	3.413 (3)	133
N2—H2*D*⋯Cl4^iii^	0.90	2.74	3.497 (3)	142

Symmetry codes: (i) 
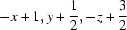
; (ii) 
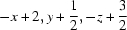
; (iii) 
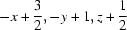
; (iv) 
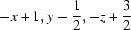
; (v) 
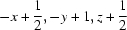
.

**Fig. 1.**
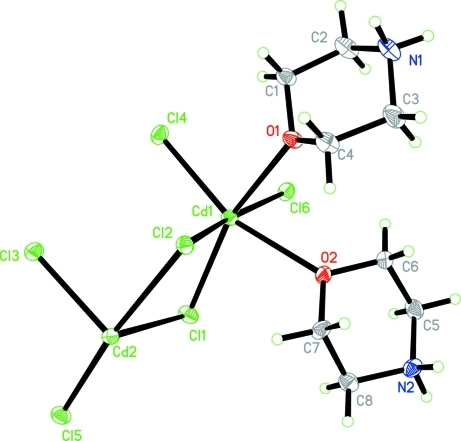
The molecular structure of the title compound, with the displacement ellipsoids drawn at the 30% probability level.

**Fig. 2.**
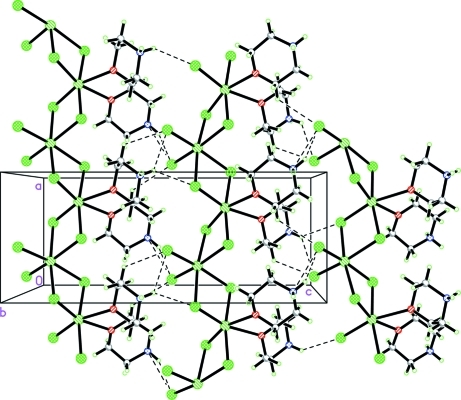
View along the c axis of the packing arrangement and intermolecular hydrogen bonds for the title compound.

## supplementary crystallographic information

### Comment

Currently, the area of coordination polymers has undergone much development, with the aim of designing new materials with interesting physical properties. Numerous one-, two- and three-dimensional structures have been synthesized and characterized. (Xiong, *et al.*, 1999; Ye, *et al.*, 2005; Zhao *et al.*, 2008) In the present work, a reaction of MOR cations, HCl, and cadmium(II) chloride has produced a novel one-dimensional coordination polymer, in which N–H–Cl hydrogen bonds interactions aggregate the anions and cations into a two-dimensional network.

Quite different from that observed in the dimeric coordination compound of (MOR)_2_Cu_2_Cl_6_ (Willett *et al.*, 2005), which links one morphine in each copper atom forming a semi-coordinate bond. The title compound [C_8_H_20_Cd_2_N_2_O_2_Cl_6_] exhibits a new coordinated mode. It is shown that two Cd centers have quite different coordination environments. The Cd1 atom is octahedrally coordinated by four Cl atoms and two O atoms from two MOR ligands. The Cd2 atom is octahedrally coordinated by six Cl atoms. Interestingly, adjacent Cd ions are interconnected alternately by paired chloride bridges to generate an infinite one-dimensional coordination chain along the *a* axis.The compound is assembled into layer structures *via* 6 kinds of N–H—Cl synthons as shown in Fig 2. Due to the interaction of N–H—Cl hydrogen bond,from which the two protons on a given NH^2+^ group form bifurcated hydrogen bonds, the polymer constitute a two-dimensional framework at [1 1 0].

### Experimental

MOR 0.87 g(1 mmol) was dissolved in ethanol,with carefully dripping hydrochloric acid 1 g(30%). After stirring 20 min, 2.5 g(.0.85 mmol)of dissolved cadmium chloride by water was mixed. Then filtering the solution to keep it cleaning. The reaction solution was cooled down to room temperature to volatilization. Colorless needlelike crystals was obtained on the tube wall after three days and of average size 0.13 *x* 0.28 *x* 0.42 mm

### Refinement

Positional parameters of all the H atoms were calculated geometrically and were allowed to ride on the C, N atoms to which they are bonded, with C—H =0.97 Å, *U*_iso_(H) = 1.2 *U*_eq_(C), N—H = 0.90 Å, *U*_iso_(H)= 1.5 *U*_eq_(N).

### Crystal data

**Table:**

[Cd_2_Cl_6_(C_4_H_10_NO)_2_]	*F*(000) = 1184
*M**_r_* = 613.78	*D*_x_ = 2.283 Mg m^−^^3^
Orthorhombic, *P*2_1_2_1_2_1_	Mo *K*α radiation, λ = 0.71073 Å
Hall symbol: p 2ac 2ab	Cell parameters from 1977 reflections
*a* = 7.0496 (14) Å	θ = 3.1–27.5°
*b* = 14.404 (3) Å	µ = 3.28 mm^−^^1^
*c* = 17.583 (4) Å	*T* = 298 K
*V* = 1785.4 (7) Å^3^	Needle, colourless
*Z* = 4	0.45 × 0.30 × 0.15 mm

### Data collection

**Table:**

Rigaku SCXmini diffractometer	4100 independent reflections
Radiation source: fine-focus sealed tube	3893 reflections with *I* > 2σ(*I*)
graphite	*R*_int_ = 0.035
Detector resolution: 13.6612 pixels mm^-1^	θ_max_ = 27.5°, θ_min_ = 3.1°
CCD_Profile_fitting scans	*h* = −9→9
Absorption correction: multi-scan (*CrystalClear*; Rigaku, 2005)	*k* = −18→18
*T*_min_ = 0.319, *T*_max_ = 0.611	*l* = −22→22
18533 measured reflections	

### Refinement

**Table:**

Refinement on *F*^2^	Primary atom site location: structure-invariant direct methods
Least-squares matrix: full	Secondary atom site location: difference Fourier map
*R*[*F*^2^ > 2σ(*F*^2^)] = 0.023	Hydrogen site location: inferred from neighbouring sites
*wR*(*F*^2^) = 0.051	H-atom parameters constrained
*S* = 1.07	*w* = 1/[σ^2^(*F*_o_^2^) + (0.0224*P*)^2^ + 0.3409*P*] where *P* = (*F*_o_^2^ + 2*F*_c_^2^)/3
4100 reflections	(Δ/σ)_max_ = 0.003
181 parameters	Δρ_max_ = 0.31 e Å^−^^3^
0 restraints	Δρ_min_ = −0.61 e Å^−^^3^

### Special details

**Table:**

Geometry. All e.s.d.'s (except the e.s.d. in the dihedral angle between two l.s. planes) are estimated using the full covariance matrix. The cell e.s.d.'s are taken into account individually in the estimation of e.s.d.'s in distances, angles and torsion angles; correlations between e.s.d.'s in cell parameters are only used when they are defined by crystal symmetry. An approximate (isotropic) treatment of cell e.s.d.'s is used for estimating e.s.d.'s involving l.s. planes.
Refinement. Refinement of *F*^2^ against ALL reflections. The weighted *R*-factor *wR* and goodness of fit *S* are based on *F*^2^, conventional *R*-factors *R* are based on *F*, with *F* set to zero for negative *F*^2^. The threshold expression of *F*^2^ > σ(*F*^2^) is used only for calculating *R*-factors(gt) *etc*. and is not relevant to the choice of reflections for refinement. *R*-factors based on *F*^2^ are statistically about twice as large as those based on *F*, and *R*- factors based on ALL data will be even larger.

### Fractional atomic coordinates and isotropic or equivalent isotropic displacement parameters (Å^2^)

**Table:**

	*x*	*y*	*z*	*U*_iso_*/*U*_eq_	
Cd1	0.72480 (3)	0.507712 (15)	0.684293 (13)	0.02333 (6)	
Cd2	0.23018 (3)	0.497301 (15)	0.589235 (12)	0.02517 (6)	
Cl2	0.41966 (11)	0.59933 (5)	0.69463 (4)	0.02457 (16)	
Cl4	0.90345 (12)	0.60740 (6)	0.58524 (5)	0.03148 (19)	
Cl6	1.03220 (11)	0.42036 (6)	0.70226 (5)	0.03077 (19)	
Cl3	0.37109 (12)	0.60596 (6)	0.49228 (5)	0.03157 (19)	
Cl1	0.53066 (12)	0.38288 (6)	0.61612 (5)	0.03188 (19)	
O1	0.8396 (3)	0.61772 (16)	0.78576 (12)	0.0280 (5)	
N1	1.0415 (4)	0.7015 (2)	0.90750 (17)	0.0360 (7)	
H1A	1.0127	0.7623	0.9054	0.043*	
H1B	1.1214	0.6928	0.9468	0.043*	
C4	0.7403 (5)	0.6518 (2)	0.85098 (19)	0.0347 (8)	
H4A	0.6266	0.6151	0.8590	0.042*	
H4B	0.7023	0.7156	0.8423	0.042*	
C3	0.8640 (6)	0.6466 (3)	0.9206 (2)	0.0425 (10)	
H3A	0.7963	0.6714	0.9641	0.051*	
H3B	0.8960	0.5824	0.9313	0.051*	
C2	1.1364 (5)	0.6735 (3)	0.8358 (2)	0.0386 (9)	
H2A	1.1879	0.6114	0.8414	0.046*	
H2B	1.2408	0.7155	0.8254	0.046*	
C1	0.9997 (5)	0.6753 (3)	0.77094 (18)	0.0325 (8)	
H1C	0.9576	0.7385	0.7624	0.039*	
H1D	1.0629	0.6539	0.7252	0.039*	
Cl5	0.10021 (12)	0.37744 (6)	0.49674 (5)	0.03176 (19)	
O2	0.6243 (3)	0.43396 (16)	0.80860 (13)	0.0319 (6)	
C8	0.3919 (5)	0.3182 (2)	0.8424 (2)	0.0315 (8)	
H8A	0.2587	0.3089	0.8541	0.038*	
H8B	0.4234	0.2804	0.7985	0.038*	
C7	0.4274 (4)	0.4181 (2)	0.8249 (2)	0.0318 (8)	
H7A	0.3514	0.4365	0.7815	0.038*	
H7B	0.3895	0.4559	0.8680	0.038*	
C6	0.7363 (5)	0.4118 (2)	0.87368 (17)	0.0316 (8)	
H6A	0.6979	0.4506	0.9161	0.038*	
H6B	0.8687	0.4246	0.8629	0.038*	
N2	0.5099 (4)	0.28953 (19)	0.90858 (16)	0.0300 (7)	
H2C	0.4962	0.2282	0.9165	0.036*	
H2D	0.4699	0.3194	0.9505	0.036*	
C5	0.7142 (5)	0.3111 (2)	0.8952 (2)	0.0351 (8)	
H5A	0.7629	0.2720	0.8547	0.042*	
H5B	0.7865	0.2982	0.9410	0.042*	

### Atomic displacement parameters (Å^2^)

**Table:**

	*U*^11^	*U*^22^	*U*^33^	*U*^12^	*U*^13^	*U*^23^
Cd1	0.01729 (11)	0.02593 (12)	0.02677 (12)	0.00002 (11)	0.00033 (8)	0.00048 (10)
Cd2	0.02531 (12)	0.02594 (12)	0.02425 (12)	−0.00506 (12)	−0.00041 (8)	0.00121 (10)
Cl2	0.0224 (4)	0.0255 (4)	0.0258 (4)	0.0022 (3)	−0.0006 (3)	0.0002 (3)
Cl4	0.0300 (4)	0.0365 (5)	0.0280 (4)	0.0000 (4)	0.0046 (3)	0.0075 (4)
Cl6	0.0213 (4)	0.0357 (4)	0.0353 (5)	0.0040 (3)	0.0011 (3)	0.0101 (4)
Cl3	0.0338 (5)	0.0352 (5)	0.0256 (4)	−0.0084 (4)	0.0023 (3)	0.0036 (4)
Cl1	0.0281 (4)	0.0280 (4)	0.0395 (5)	−0.0002 (3)	−0.0061 (4)	−0.0057 (4)
O1	0.0246 (12)	0.0347 (13)	0.0246 (12)	−0.0051 (10)	0.0029 (9)	−0.0076 (10)
N1	0.0292 (16)	0.0423 (18)	0.0365 (17)	0.0039 (14)	−0.0070 (14)	−0.0152 (15)
C4	0.0261 (19)	0.045 (2)	0.0327 (18)	−0.0024 (17)	0.0074 (16)	−0.0129 (15)
C3	0.045 (2)	0.053 (3)	0.029 (2)	−0.007 (2)	0.0011 (17)	−0.0127 (18)
C2	0.0267 (19)	0.051 (2)	0.038 (2)	−0.0008 (17)	0.0012 (16)	−0.0165 (18)
C1	0.0271 (19)	0.043 (2)	0.027 (2)	−0.0098 (16)	0.0036 (15)	−0.0054 (15)
Cl5	0.0317 (4)	0.0331 (4)	0.0305 (4)	−0.0060 (4)	−0.0034 (4)	−0.0049 (4)
O2	0.0229 (12)	0.0453 (14)	0.0276 (13)	−0.0066 (11)	−0.0028 (10)	0.0131 (11)
C8	0.0253 (18)	0.035 (2)	0.034 (2)	0.0018 (15)	−0.0012 (15)	−0.0027 (16)
C7	0.0211 (17)	0.039 (2)	0.0357 (19)	0.0013 (15)	0.0048 (14)	0.0137 (16)
C6	0.032 (2)	0.0362 (18)	0.0265 (18)	−0.0057 (16)	−0.0049 (15)	0.0055 (13)
N2	0.0386 (18)	0.0201 (14)	0.0312 (16)	−0.0014 (13)	0.0028 (13)	0.0020 (12)
C5	0.036 (2)	0.0305 (18)	0.039 (2)	0.0028 (17)	−0.0134 (17)	0.0035 (15)

### Geometric parameters (Å, °)

**Table:**

Cd1—O1	2.520 (2)	C3—H3A	0.9700
Cd1—Cl6	2.5257 (9)	C3—H3B	0.9700
Cd1—Cl2	2.5301 (9)	C2—C1	1.494 (5)
Cd1—O2	2.531 (2)	C2—H2A	0.9700
Cd1—Cl1	2.5579 (9)	C2—H2B	0.9700
Cd1—Cl4	2.5848 (9)	C1—H1C	0.9700
Cd2—Cl3	2.5184 (9)	C1—H1D	0.9700
Cd2—Cl5	2.5427 (9)	O2—C6	1.427 (4)
Cd2—Cl6^i^	2.6694 (9)	O2—C7	1.436 (4)
Cd2—Cl2	2.7163 (9)	C8—N2	1.489 (4)
Cd2—Cl1	2.7251 (10)	C8—C7	1.493 (5)
Cd2—Cl4^i^	2.7974 (10)	C8—H8A	0.9700
Cl4—Cd2^ii^	2.7974 (10)	C8—H8B	0.9700
Cl6—Cd2^ii^	2.6694 (9)	C7—H7A	0.9700
O1—C1	1.424 (4)	C7—H7B	0.9700
O1—C4	1.430 (4)	C6—C5	1.508 (4)
N1—C2	1.482 (4)	C6—H6A	0.9700
N1—C3	1.498 (5)	C6—H6B	0.9700
N1—H1A	0.9000	N2—C5	1.492 (5)
N1—H1B	0.9000	N2—H2C	0.9000
C4—C3	1.505 (5)	N2—H2D	0.9000
C4—H4A	0.9700	C5—H5A	0.9700
C4—H4B	0.9700	C5—H5B	0.9700
			
O1—Cd1—Cl6	87.09 (6)	N1—C3—H3A	109.8
O1—Cd1—Cl2	83.92 (5)	C4—C3—H3A	109.8
Cl6—Cd1—Cl2	168.61 (3)	N1—C3—H3B	109.8
O1—Cd1—O2	75.07 (7)	C4—C3—H3B	109.8
Cl6—Cd1—O2	85.59 (6)	H3A—C3—H3B	108.2
Cl2—Cd1—O2	85.34 (6)	N1—C2—C1	110.7 (3)
O1—Cd1—Cl1	161.03 (5)	N1—C2—H2A	109.5
Cl6—Cd1—Cl1	99.64 (3)	C1—C2—H2A	109.5
Cl2—Cd1—Cl1	86.87 (3)	N1—C2—H2B	109.5
O2—Cd1—Cl1	87.70 (6)	C1—C2—H2B	109.5
O1—Cd1—Cl4	88.36 (6)	H2A—C2—H2B	108.1
Cl6—Cd1—Cl4	86.73 (3)	O1—C1—C2	111.2 (3)
Cl2—Cd1—Cl4	99.96 (3)	O1—C1—H1C	109.4
O2—Cd1—Cl4	162.05 (6)	C2—C1—H1C	109.4
Cl1—Cd1—Cl4	109.60 (3)	O1—C1—H1D	109.4
Cl3—Cd2—Cl5	97.53 (3)	C2—C1—H1D	109.4
Cl3—Cd2—Cl6^i^	165.51 (3)	H1C—C1—H1D	108.0
Cl5—Cd2—Cl6^i^	90.34 (3)	C6—O2—C7	109.9 (2)
Cl3—Cd2—Cl2	86.08 (3)	C6—O2—Cd1	129.16 (18)
Cl5—Cd2—Cl2	168.80 (3)	C7—O2—Cd1	120.65 (18)
Cl6^i^—Cd2—Cl2	88.50 (3)	N2—C8—C7	109.5 (3)
Cl3—Cd2—Cl1	100.76 (3)	N2—C8—H8A	109.8
Cl5—Cd2—Cl1	88.88 (3)	C7—C8—H8A	109.8
Cl6^i^—Cd2—Cl1	91.50 (3)	N2—C8—H8B	109.8
Cl2—Cd2—Cl1	80.02 (3)	C7—C8—H8B	109.8
Cl3—Cd2—Cl4^i^	87.45 (3)	H8A—C8—H8B	108.2
Cl5—Cd2—Cl4^i^	94.14 (3)	O2—C7—C8	110.9 (3)
Cl6^i^—Cd2—Cl4^i^	79.84 (3)	O2—C7—H7A	109.5
Cl2—Cd2—Cl4^i^	96.62 (3)	C8—C7—H7A	109.5
Cl1—Cd2—Cl4^i^	170.83 (3)	O2—C7—H7B	109.5
Cd1—Cl2—Cd2	94.98 (3)	C8—C7—H7B	109.5
Cd1—Cl4—Cd2^ii^	93.98 (3)	H7A—C7—H7B	108.1
Cd1—Cl6—Cd2^ii^	98.55 (3)	O2—C6—C5	111.1 (3)
Cd1—Cl1—Cd2	94.13 (3)	O2—C6—H6A	109.4
C1—O1—C4	109.6 (2)	C5—C6—H6A	109.4
C1—O1—Cd1	119.39 (18)	O2—C6—H6B	109.4
C4—O1—Cd1	128.78 (19)	C5—C6—H6B	109.4
C2—N1—C3	111.4 (3)	H6A—C6—H6B	108.0
C2—N1—H1A	109.4	C8—N2—C5	111.0 (3)
C3—N1—H1A	109.4	C8—N2—H2C	109.4
C2—N1—H1B	109.4	C5—N2—H2C	109.4
C3—N1—H1B	109.4	C8—N2—H2D	109.4
H1A—N1—H1B	108.0	C5—N2—H2D	109.4
O1—C4—C3	110.6 (3)	H2C—N2—H2D	108.0
O1—C4—H4A	109.5	N2—C5—C6	109.8 (3)
C3—C4—H4A	109.5	N2—C5—H5A	109.7
O1—C4—H4B	109.5	C6—C5—H5A	109.7
C3—C4—H4B	109.5	N2—C5—H5B	109.7
H4A—C4—H4B	108.1	C6—C5—H5B	109.7
N1—C3—C4	109.5 (3)	H5A—C5—H5B	108.2
			
O1—Cd1—Cl2—Cd2	−178.93 (5)	Cl4—Cd1—O1—C1	−20.0 (2)
Cl6—Cd1—Cl2—Cd2	−140.85 (12)	Cl6—Cd1—O1—C4	−132.1 (3)
O2—Cd1—Cl2—Cd2	−103.48 (6)	Cl2—Cd1—O1—C4	40.9 (3)
Cl1—Cd1—Cl2—Cd2	−15.53 (3)	O2—Cd1—O1—C4	−45.9 (3)
Cl4—Cd1—Cl2—Cd2	93.82 (3)	Cl1—Cd1—O1—C4	−20.5 (4)
Cl3—Cd2—Cl2—Cd1	−86.89 (3)	Cl4—Cd1—O1—C4	141.1 (3)
Cl5—Cd2—Cl2—Cd1	22.39 (15)	C1—O1—C4—C3	−63.0 (4)
Cl6^i^—Cd2—Cl2—Cd1	106.55 (3)	Cd1—O1—C4—C3	134.4 (3)
Cl1—Cd2—Cl2—Cd1	14.76 (3)	C2—N1—C3—C4	−52.2 (4)
Cl4^i^—Cd2—Cl2—Cd1	−173.86 (2)	O1—C4—C3—N1	57.9 (4)
O1—Cd1—Cl4—Cd2^ii^	94.39 (6)	C3—N1—C2—C1	51.6 (4)
Cl6—Cd1—Cl4—Cd2^ii^	7.21 (3)	C4—O1—C1—C2	62.0 (4)
Cl2—Cd1—Cl4—Cd2^ii^	177.92 (2)	Cd1—O1—C1—C2	−133.6 (2)
O2—Cd1—Cl4—Cd2^ii^	72.00 (19)	N1—C2—C1—O1	−56.4 (4)
Cl1—Cd1—Cl4—Cd2^ii^	−91.87 (3)	O1—Cd1—O2—C6	−53.8 (2)
O1—Cd1—Cl6—Cd2^ii^	−96.14 (6)	Cl6—Cd1—O2—C6	34.3 (2)
Cl2—Cd1—Cl6—Cd2^ii^	−134.03 (12)	Cl2—Cd1—O2—C6	−138.8 (2)
O2—Cd1—Cl6—Cd2^ii^	−171.38 (6)	Cl1—Cd1—O2—C6	134.2 (2)
Cl1—Cd1—Cl6—Cd2^ii^	101.71 (3)	Cl4—Cd1—O2—C6	−30.6 (4)
Cl4—Cd1—Cl6—Cd2^ii^	−7.62 (3)	O1—Cd1—O2—C7	118.8 (2)
O1—Cd1—Cl1—Cd2	76.43 (18)	Cl6—Cd1—O2—C7	−153.1 (2)
Cl6—Cd1—Cl1—Cd2	−173.94 (3)	Cl2—Cd1—O2—C7	33.9 (2)
Cl2—Cd1—Cl1—Cd2	15.46 (3)	Cl1—Cd1—O2—C7	−53.2 (2)
O2—Cd1—Cl1—Cd2	100.92 (6)	Cl4—Cd1—O2—C7	142.0 (2)
Cl4—Cd1—Cl1—Cd2	−84.00 (3)	C6—O2—C7—C8	−62.2 (4)
Cl3—Cd2—Cl1—Cd1	69.44 (3)	Cd1—O2—C7—C8	123.8 (2)
Cl5—Cd2—Cl1—Cd1	166.90 (3)	N2—C8—C7—O2	58.5 (4)
Cl6^i^—Cd2—Cl1—Cd1	−102.80 (3)	C7—O2—C6—C5	61.0 (4)
Cl2—Cd2—Cl1—Cd1	−14.58 (3)	Cd1—O2—C6—C5	−125.7 (3)
Cl6—Cd1—O1—C1	66.8 (2)	C7—C8—N2—C5	−54.2 (4)
Cl2—Cd1—O1—C1	−120.2 (2)	C8—N2—C5—C6	53.1 (4)
O2—Cd1—O1—C1	153.0 (2)	O2—C6—C5—N2	−56.5 (4)
Cl1—Cd1—O1—C1	178.43 (19)		

Symmetry codes: (i) *x*−1, *y*, *z*; (ii) *x*+1, *y*, *z*.

### Hydrogen-bond geometry (Å, °)

**Table:**

*D*—H···*A*	*D*—H	H···*A*	*D*···*A*	*D*—H···*A*
N1—H1A···Cl5^iii^	0.90	2.52	3.203 (3)	133.
N1—H1A···Cl6^iv^	0.90	2.98	3.733 (4)	143.
N1—H1B···Cl5^v^	0.90	2.38	3.183 (3)	149.
N2—H2C···Cl3^vi^	0.90	2.56	3.276 (3)	137.
N2—H2C···Cl2^vi^	0.90	2.76	3.323 (3)	122.
N2—H2D···Cl3^vii^	0.90	2.73	3.413 (3)	133.
N2—H2D···Cl4^v^	0.90	2.74	3.497 (3)	142.

Symmetry codes: (iii) −*x*+1, *y*+1/2, −*z*+3/2; (iv) −*x*+2, *y*+1/2, −*z*+3/2; (v) −*x*+3/2, −*y*+1, *z*+1/2; (vi) −*x*+1, *y*−1/2, −*z*+3/2; (vii) −*x*+1/2, −*y*+1, *z*+1/2.
